# 20-Hydroxyecdysone Confers Antioxidant and Antineoplastic Properties in Human Non-Small Cell Lung Cancer Cells

**DOI:** 10.3390/metabo13050656

**Published:** 2023-05-15

**Authors:** Oleg Shuvalov, Yulia Kirdeeva, Elizaveta Fefilova, Sofia Netsvetay, Mark Zorin, Yulia Vlasova, Olga Fedorova, Alexandra Daks, Sergey Parfenyev, Nickolai Barlev

**Affiliations:** 1 Institute of Cytology, Russian Academy of Sciences, 194064 St. Petersburg, Russia; yulia.kirdeeva@yandex.ru (Y.K.); e.fefilova@list.ru (E.F.); sofya.necvetaj@spcpu.ru (S.N.); fedorovaolga0402@gmail.com (O.F.); gen21eration@gmail.com (S.P.); 2 Almazov National Medical Research Center Russia, 197341 St. Petersburg, Russia; vlasovayu@rambler.ru; 3 School of Medicine, Nazarbayev University, 001000 Astana, Kazakhstan; 4 Institute of Fundamental Medicine and Biology, Kazan Federal University, 420008 Kazan, Russia

**Keywords:** 20-hydroxyecdysone, non-small cell lung cancer, glycolysis, respiration, energy metabolism, one-carbon metabolism, cancer stem cells (CSCs), metabolic inhibitors, anticancer therapy

## Abstract

20-Hydroxyecdysone (20E) is an arthropod hormone which is synthesized by some plants as part of their defense mechanism. In humans, 20E has no hormonal activity but possesses a number of beneficial pharmacological properties including anabolic, adaptogenic, hypoglycemic, and antioxidant properties, as well as cardio-, hepato-, and neuroprotective features. Recent studies have shown that 20E may also possess antineoplastic activity. In the present study, we reveal the anticancer properties of 20E in Non-Small Cell Lung Cancer (NSCLC) cell lines. 20E displayed significant antioxidant capacities and induced the expression of antioxidative stress response genes. The RNA-seq analysis of 20E-treated lung cancer cells revealed the attenuation of genes involved in different metabolic processes. Indeed, 20E suppressed several enzymes of glycolysis and one-carbon metabolism, as well as their key transcriptional regulators—c-Myc and ATF4, respectively. Accordingly, using the SeaHorse energy profiling approach, we observed the inhibition of glycolysis and respiration mediated by 20E treatment. Furthermore, 20E sensibilized lung cancer cells to metabolic inhibitors and markedly suppressed the expression of Cancer Stem Cells (CSCs) markers. Thus, in addition to the known beneficial pharmacological activities of 20E, our data uncovered novel antineoplastic properties of 20E in NSCLC cells.

## 1. Introduction

20-Hydroxyecdysone (20E) is a natural sterol compound, and a hormone in invertebrates (particularly insects). It can also occur in some plant species, where it seems to assist in the plant’s defense from its invertebrate feeders. This natural compound attracts a lot of attention due to its beneficial pharmacologic properties in humans. 20E displays anabolic, hypolipidemic, anti-diabetic, anti-inflammatory, hepato- and cardioprotective, antioxidant, antihypertensive, anti-fibrotic, and anti-COVID properties, among others [1,2,3,4]. It is also extensively used as an anabolic and adaptogenic substance in the form of dietary supplements. 

The 20E compound displays low toxicity. The LD50 of 20E in mice is 9 g/kg of weight [5], whereas the dose recommended for sports enhancement is 500–1000 mg a day. 20E is a major biologically active constituent of *Leuzea cartamoides* (“Maral rute”)—the traditional medical plant of the Altai region (Russia), where it has been used as an adaptogenic therapeutic [6]. In China, 20E is extracted from another plant—*Cyanotis arachnoideae*—which is now the main source of commercially available 20E in the form of bioactive supplements worldwide [7]. Additionally, it is now possible to purify 20E from plants with a 98% purity, due to the availability of standardized protocols [1]. 

Pharmacokinetic studies of 20E in mice and humans have been conducted [8,9]. Clinical investigations have shown that this compound demonstrates a safe profile [1]. Moreover, the BioPhytis company (France) has carried out several clinical trials of 20E to treat Dushen’s myodystrophy, Alzheimer’s disease, Sarcopenia, and severe distress syndrome as a consequence of COVID-19 incidence (https://www.biophytis.com/, accessed on 15 February 2023) [1,3]. The last trial is currently ongoing as a Phase 3 study. The molecular mechanisms of 20E activity are likely linked to its ability to impact the renin–angiotensin system [10] and Estrogen Receptor beta (ER-β) [11].

Several studies have reported on the potential antitumor properties of 20E. We and others have shown that 20E and its derivates sensibilize breast cancer cells to genotoxic stress, induce autophagy, and attenuate multiple drug resistance [12,13,14,15,16,17,18,19].

Metabolic rewiring, including different metabolic alterations, is considered to be one of the “hallmarks of cancer” [20]. There are a lot of ongoing efforts to utilize the process of metabolic reprogramming in Non-Small Cell Lung Cancer (NSCLC) as a therapeutic opportunity [21,22,23,24]. 

In the present study, we investigated the anticancer properties of 20E in several cell models of NSCLC cell lines. RNA-seq analysis revealed an inhibitory role of 20E towards a large number of genes with oncogenic properties in lung cancer. We observed strong antioxidant capacities, as well as an inhibitory effect of 20E on cancer-associated metabolic rewiring. 20E was able to sensitize lung cancer cells to metabolic inhibitors and suppress the expression of genes which are considered as markers of Cancer Stem Cells (CSCs).

## 2. Materials and Methods

### 2.1. Cell Lines and Reagents

The NSCLC cell lines used in this study (A549, H1299, and H460) were purchased from ATCC. They were cultured in a DMEM with a low glucose concentration (1 mg/mL), supplemented with 10% FBS, 100 μg/mL gentamycin, and 2 mM L-glutamine at 37 °C in a 5% CO_2_ atmosphere.

20E (ecdysterone), (BioSynth, San Diego, CA, USA, 95% purity) was dissolved in DMSO. DMSO was used as a control for all experiments with 20E. 2-DG (2-deoxyglucose, (Sigma, St. Louis, MO, USA) 98% purity), 3-BP (3-Bromopyruvate, (Sigma, MO, USA), 98% purity), gemcitabine (Teva, Tel Aviv, Israel, 95% purity), and metformin (Sigma, MO, USA, 98% purity) were dissolved in water.

### 2.2. Measurement of ROS Level

The total level of reactive oxygen species (ROS) was analyzed using 2′,7′-dichlorodihydrofluorescein diacetate (H_2_DCFDA, Invitrogen, Waltham, MA USA). Detached with trypsin, and resuspended, cells were treated with 50 µM H_2_DCFDA for 40 min at 37 °C in a CO_2_ incubator and analyzed using flow cytometry (CytoFlex, Beckman Coulter, Carlsbad, CA, USA). Results are represented as the mean ± SD of three experiments.

### 2.3. Real-Time PCR

Total RNA was extracted from cells using a TRIzol-based Reagent (Evrogene, Moscow, Russia) and following the manufacturer’s instructions. Three micrograms of total RNA were used for reverse transcription with oligo d(T) primer using a RevertAid First-Strand cDNA Synthesis Kit (Evrogen, Russia). Real-time PCR was performed using a CFX 1000 PCR machine (BioRad, Hercules, CA, USA) using SYBR green mix (Evrogen, Russia) in triplicates. Data were analyzed with CFX Manager software. β-actin was used as a reference. Relative expression was calculated using 2^−∆∆Ct^ method. Sequences of primers are listed in Table S1. 

### 2.4. Qiagen RT^2^ Profiler™ PCR Array

To profile the expression of a panel of genes associated with antioxidant response, the H460 and H1299 cells were treated with 10 and 1 µM of 20E, respectively, for 24 h. Then, RNA was extracted and cDNA synthesis was performed as described in the previous section. To profile gene expression, Qiagen RT^2^ Profiler™ PCR Array Human Oxidative Stress kit was used in accordance with the manufacturer’s instructions. QiaGen Globe online software (https://geneglobe.qiagen.com/) was applied to analyze the results obtained. GraphPad software (https://www.graphpad.com/features) was used for heatmap construction.

### 2.5. RNA-Seq Analysis

For RNA extraction, the RNA Solo Kit (Evrogen, Russia) was used during the DNase I treatment step in accordance with the manufacturer’s recommendations. RNA quality and concentration were evaluated on a 2100 Bioanalyzer (Agilent Technologies, Santa Clara, CA, USA) with an Agilent RNA 6000 Nano Kit (Agilent Technologies, USA), and on a Qubit 2.0 (Life Technologies, Carlsbad, CA, USA) with a Qubit RNA BR Assay Kit (Life Technologies, Carlsbad, CA, USA), respectively.

For cDNA library preparation, 1 μg of total RNA, the NEBNext Poly(A) mRNA Magnetic Isolation Module (New England Biolabs, Hitchin, UK), and NEBNext Ultra II Directional RNA Library Prep Kit for Illumina (New England Biolabs) were used. The cDNA library quality and concentration were evaluated on a 2100 Bioanalyzer (Agilent Technologies, Santa Clara, CA, USA) with an Agilent DNA 1000 Kit (Agilent Technologies, USA), and on a Qubit 2.0 (Life Technologies, USA) with a Qubit dsDNA HS Assay Kit (Life Technologies, Carlsbad, CA, USA), respectively. Transcriptome sequencing was performed on NextSeq 500 (Illumina, San Diego, CA, USA) with a read length of 86 bp.

The quality control analysis of raw single-end reads was performed using FastQC v.0.11.9 (https://www.bioinformatics.babraham.ac.uk/projects/fastqc/). Then, reads were trimmed using Trimmomatic v.0.39 (http://www.usadellab.org/cms/?page=trimmomatic) with HEADCROP:10 and CROP:60 parameters. After trimming, the contamination check analysis was performed using FastQ Screen v.0.15.1 (https://www.bioinformatics.babraham.ac.uk/projects/fastq_screen/) software and reference genomes: *Escherichia coli* strain K-12 (ASM584v2), *Homo sapience* (GRCh38.p14), *Metamycoplasma orale* strain NCTC10112 (50465_D02-3), *Mus musculus* (GRCm39), and *Staphylococcus aureus* (ASM1342v1). During the contamination check, Bowtie2 v.2.4.5 (https://bowtie-bio.sourceforge.net/bowtie2/index.shtml) was used as an aligner. Reads that did not match to any genome or align to *E. coli*, *M. orale* or *S. aureus* were excluded from subsequent analyses. Then, we aligned the filtered reads to the reference human genome (GRCh38.p14) using Hisat2 v.2.2.1 software (http://daehwankimlab.github.io/hisat2/download/), and sorted and recorded them in *bam* format using Samtools v.1.16.1 (http://www.htslib.org/). To quantify the number of reads that mapped to each gene, featureCounts v.2.0.1 was used. In the following analysis, we used only genes with more than 5 mapped reads. After filtering files, differential expression analysis using DESeq2 v.1.36.0 (https://s3.jcloud.sjtu.edu.cn/899a892efef34b1b944a19981040f55b-oss01/bioconductor/3.15/bioc/html/DESeq2.html) was performed. Under further analysis, only genes with an adjusted p-value of less than 0.05 were included.

Gene clusterization, which predicts a number of molecular processes and diseases which may be affected by 20E, was performed using DAVID online free software (https://david.ncifcrf.gov/). *p*-values indicate Fisher’s Exact *p*-values. For a detailed description of statistical tests used by David, please see https://david.ncifcrf.gov/content.jsp?file=functional_annotation.html.

### 2.6. Western-Blot

Cells were lysed using a RIPA buffer (150 mM NaCl; 50 mM Tris–HCl pH 7.5; 0.5%NP-40; 1 mM PMSF, protease inhibitor cocktail) with sonication. Then, the total protein level was quantified using BCA assay (ThermoScientific, Waltham, MA, USA) and diluted with a Laemli buffer. Then, 30 ug of protein lysate samples were run in 13% SDS-PAGE (TRIS-Glycine running buffer), followed by a transfer to a PVDF membrane (Bio-RAD, Hercules, CA, USA). Membranes were blocked with PBST-diluted 5% nonfat milk and incubated with primary antibodies: HK2 (MA5-14849, ThermoScientific, Waltham, MA, USA), LDHA (#2012, Cell Signaling, Danvers, MA, USA), c-Myc (D84C12, Cell Signaling, Danvers, MA, USA), ATF4 (DF6008, Cloud-Clone, Wuhan, China), SHMT2 (DF6347, Cloud-Clone, Wuhan, China), MTHFD2 (DF12213, Cloud-Clone, Wuhan, China), β-actin (#8457, Cell Signaling, Danvers, MA, USA). After washing several times with PBST, secondary anti-mouse or anti-rabbit antibodies (1:10,000; Sigma-Aldrich, MO, USA) conjugated with horseradish peroxidase were applied. An ECL system (ThermoScientific, Waltham, MA, USA) and ChemiDoc Touch Imager (Bio-Rad, Hercules, CA, USA) were used for detection.

The quantification was carried out using Image J software. First, the ratio of each sample/control sample was calculated for actin and other proteins. Finally, the protein/actin ratio was calculated and listed in the figure.

### 2.7. SeaHorse Energy Profiling

To study glycolysis and respiration, a Seahorse XFe24 Analyzer was used. The energy profiling of H460 treated with 20E was performed with a MitoStress test kit (Agilent Technologies, Santa Clara, CA, USA), as described in [25]. H1299 and A549 cells treated with 20E were analyzed using an Energy Phenotype test kit (Agilent Technologies, Santa Clara, CA, USA) in accordance with the manufacturer’s instructions. The final concentrations of oligomycin, FCCP and Rotenone/Antimycin A were 3, 2, and 2 µM, respectively. Results are presented as the mean ± SEM.

### 2.8. ATP Production Assay

To quantify the level of ATP, 100,000 cells per well were seeded on a 12-well plate and treated with different concentrations of 20E for 48 h. On the day of analysis, an ATP Assay Kit (AbCam, ab83355, Waltham, Boston, MA, USA) was applied according to the manufacturer’s instructions. The experiment was carried out in triplicate. Results are presented as percentage of ATP level relative to control (DMSO-treated cells).

### 2.9. MTT Assay

A day before treatment, 4000 cells were planted in each well of a 96-well plate. Ten wells per sample were used. A day after, 20E or (and) 2-DG, metformin, and gemcitabine of 5-FU were added in the required concentrations for 48–72 h. DMSO was used as a control for cells treated with 20E. On the day of analysis, 10 μL of 5 mg/mL Thiazolyl Blue (Paneko, Moscow, Russia) solution was added to each well and cells were kept for 3 h at 37 °C in a CO_2_ incubator. After removing the thiazol-containing medium, 150 μL isopropyl alcohol (supplemented with 40 mM HCl and 0.1% NP-40) was added to dissolve the MTT-formazan salt. The absorbance at 570 and 630 nm (reference) was measured using a BioRad iMark microplate reader (BioRad, Hercules, CA, USA). Results are presented as the mean ± SD.

### 2.10. Analysis of Drug Synergy

IC50 and drug synergy were obtained using the results of the MTT-assay and calculated as described in [26] using CompuSyn software (http://www.combosyn.com/). Results are presented as CI (Combination Index) plots and a Table which includes values for CI. CI < 1 reflects the synergistic action of drugs.

### 2.11. Bioinformatic Analysis of Lung Cancer Patients’ Survival Rates

To check if the expression levels of HK2, LDHA, PHGDH, PSAT1, PSPH, SHMT2, MTHFD2, c-Myc, and ATF4 (CREB-2) were associated with prognosis in lung cancer patients, the online software KM Plotter (https://kmplot.com/analysis/) was used [27]. The dataset consisted of 1925 lung cancer patients. The pairwise comparison of patients with a low expression of certain genes with patients with high expression was carried out. All genes were analyzed separately. For a detailed description of statistics used, please see [27].

### 2.12. Colony-Formation Assay

To perform the colony-formation assay, 750 cells were seeded per well on a 6-well plate, in triplicates. Upon seeding procedure, the media containing 0, 0.1, 1, 10, or 100 μM 20E were added and left for 12 days. After the indicated time, cells were fixed with acetic acid/methanol (1:7, *v*/*v*) and stained with 0.5% crystal violet. Results are represented as surviving fraction calculated as the number of colonies after treatment divided by the number of seeded cells and normalized to the plating efficiency of the control cells.

The number of colonies was calculated. Results are represented as the mean ± SEM of three biological replicates.

### 2.13. Cell Cycle Analysis

A day after seeding, cells were treated with ecdysterone (0, 0.1, 1, 10 or 100 μM) for 48 h in triplicates. After harvesting, cells were washed with PBS followed by fixation in 70% ethanol at −20 °C for 1 h. The 30 min staining of DNA content was carried out by using 50 μg/mL of PI (AbCam, Waltham, Boston, USA) and 1 μg/mL RNase A (ThermoScientific, Waltham, MA, USA). Samples were analyzed with a CytoFLEX (Beckman Coulter, Carlsbad, CA, USA) flow cytometer using PE channel. Results were processed using CyteExpert software (Beckman Coulter, Carlsbad, CA, USA).

### 2.14. Annexin V Test

The analysis of apoptosis and total cell death was carried out by using annexin V-FITC/7-AAD double staining followed by flow cytometry. An Annexin V-FITC/7-AAD Apoptosis kit (BioRad, Hercules, CA, USA) was used in accordance with the manufacturer’s protocol. Cells were treated for 48 h with 20E. A minimum of 5000 cells were analyzed with a CytoFLEX (Beckman Coulter, Carlsbad, CA, USA) flow cytometer using corresponding channels in three biological replicates. Values of the median were used for calculation. Results were represented as the mean ± SEM of three experiments.

### 2.15. Study of the Effect of 20E on CSC’s Markers

The impact of 20E on markers of NSCLC CSCs was determined in two ways. At first, cells were treated with 0, 0.1, 1, 10, and 100 µM 20E for 48 h followed by the analysis of gene expression using Real-Time PCR. 

Secondly, to create more physiological conditions which favor CSCs, we established the growth of spheroids using ultra-low adhesion 10 cm cell culture dishes. Based on the recommendations of Selby et al. [28], we seeded 47,000 and 120,000 cells for H460 and H1299 cell lines, respectively, re-suspended in 10 mL of DMEM medium with a low glucose concentration (1 mg/mL), supplemented with 10% FBS, 100 μg/mL gentamycin, and 2 mM L-glutamine. The growth of spheroids was checked. After 72 h, 20E to a final concentration of 1 µM or DMSO (Control) was added for 48 h followed by Real-Time PCR for markers of CSCs. Sequences of PCR primers are listed in Table S2.

### 2.16. Statistical Analysis

In our study, one-way ANOVA with Dunnet’s multiple comparisons test post hoc in GraphPad Prism 8 was used as a statistical method. *p* < 0.05 was considered as statistically different. * *p* ≤ 0.05; ** *p* ≤ 0.01; *** *p* ≤ 0.001; **** *p* ≤ 0.0001; n.s.—non-significant.

## 3. Results

### 3.1. 20E Down-Regulates ROS Levels and Induces the Expression of Antioxidative Response Genes

Elevated levels of intracellular ROS are known to be one of the reasons for genomic instability and, hence, carcinogenesis [29]. Several published reports have written about the ROS-scavenger and antioxidant properties of 20E in several in vitro [30,31] and in vivo systems [32,33]. Thus, we decided to elucidate whether 20E possesses antioxidant properties in lung cancer cells. 

**Figure 1:**
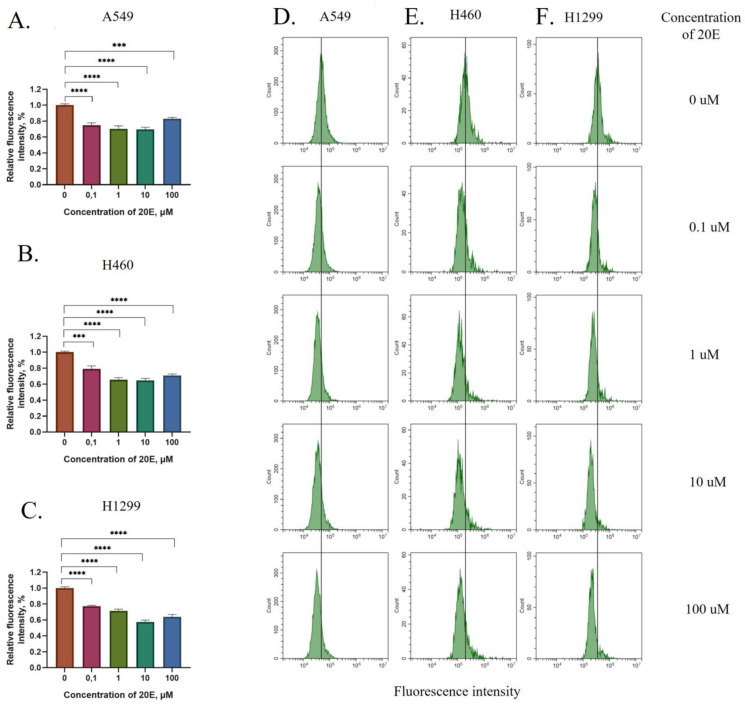
The 1.5 h treatment with 20E suppresses ROS in NSCLC cell lines. (**A**–**C**) Diagrams of relative DCDFA fluorescence for A549, H460, and H1299 cells treated with 0.1–100 µM of 20E. Y-axis shows the degree of fluorescence intensity of 20E treated cells relative to the fluorescence of control cells. (**D**–**F**) Flow cytometry plots for DCDFA fluorescence; ‘median’ of the peak for control sample is showed by vertical bar. *** *p* ≤ 0.001; **** *p* ≤ 0.0001.

First, we assessed the ability of 20E, at a broad range of concentrations, to suppress intrinsic levels of ROS in three NSCLC cell lines—H460, H1299, and A549. Cells were treated with 20E at different concentrations (0.1–100 µM) for 1.5 h. This was followed by the measurement of total levels of ROS using DCDFA staining and flow cytometry. Results shown in Figure 1 revealed a significant 20E-mediated antioxidative effect at all the concentrations tested. Thus, even 0.1 µM of 20E was able to suppress the total level of ROS by up to 20%, whereas other concentrations had inhibitory effects of up to 30–43% of the original level (Figure 1A–C, Table S2). These data suggest that 20E possesses either direct ROS scavenging activity, or that it impacts ROS indirectly, via affecting the respective signaling pathways and thereby leading to a quick antioxidant response on the level of protein–protein interactions.

Then, we decided to extend the time of the treatment to assess the duration of the 20E-mediated antioxidative effect. To this end, we treated the cells with the same 20E concentrations for 24 h. As seen from Figure S1*,* after a day of treatment ROS levels were also suppressed by 20–25%. 

**Figure 2:**
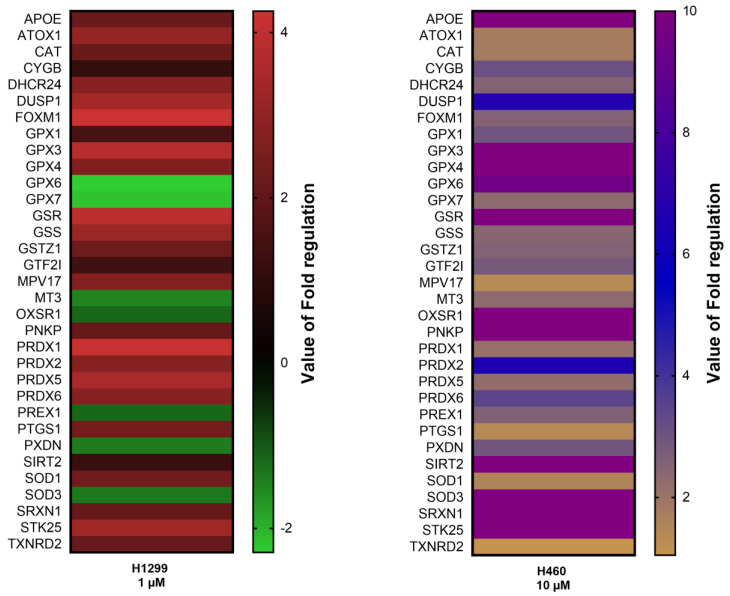
The heatmap showing the expression of genes associated with antioxidant response after the treatment of H1299 and H460 cells with 20E for 24 h (Qiagen RT² Profiler™ PCR Array Human Oxidative Stress kit). The Fold Regulations display a value of the normalized gene expression in 20E-treated cells divided by the normalized gene expression in the control (DMSO-treated) cells.

In order to elucidate whether this effect was due solely to the scavenger activity of 20E, or whether the antioxidative response enzymes were also involved, we profiled the expression of a panel of genes associated with the antioxidative stress response. For this experiment, H460 and H1299 cell lines were used. Cells were treated with 10 and 1 µM of 20E, respectively, for 24 h, followed by RT-PCR using the Qiagen RT^2^ Profiler™ PCR Array Human Oxidative Stress kit. The RT-PCR analysis revealed a number of differentially expressed genes associated with antioxidant response. Interestingly, depending on the cell line used, 20E induced the expression of different anti-oxidative genes to different levels (Figure 2): glutathione peroxidases (*Gpx3*, *Gpx4*, *Gpx6*), glutathion redustase (*Gsr*), glutathion syntetase (*Gss*), peroredoxines (*Prdx1*, *Prdx5*, *Prdx6*), superoxidismutases (*Sod1*, *Sod2*, *Sod3*), and others (Figure 2, Table S3).

Taken together, these data demonstrate that 20E can strongly decrease the level of ROS and induce an antioxidative stress response in lung cancer cells.

### 3.2. 20E Slightly Inhibits the Growth of NSCLC Cell Lines

**Figure 3:**
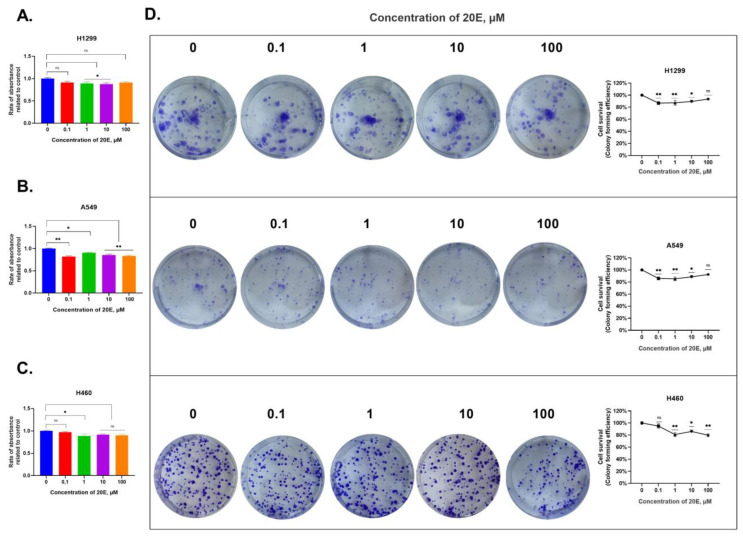
20E inhibits the growth of NSCLC cell lines. (**A**–**C**). MTT data for H1299, A549, and H460 cell lines. (**D**). Colony formation assay. * *p* ≤ 0.05; ** *p* ≤ 0.01, ns—not significant.

To elucidate whether the same range of 20E concentrations had an impact on the growth of lung cancer cells, we carried out an MTT assay. Cells were grown with 20E for 48 h. The results demonstrated in Figure 3A–C and Table S2 imply that 20E only slightly suppressed (from 10 to 15%) the growth of all cell lines. To uncover the molecular mechanism of 20E-mediated negative regulation, we carried out cell-cycle analysis and tested for apoptosis. 

According to the cell cycle analysis, no obvious changes were observed with only 100 µM of 20E in the decreased S-phase and increased G0/G1 in H1299 and A549 cells (Figures S2–S4). Moreover, the annexin-V test for apoptosis did not reveal any differences in the amount of either dead or apoptotic cells between 20E-treated and control cells (Figures S5–S7). 

### 3.3. RNA-Seq Analysis of the 20E-Mediated Impact on Gene Expression

To further elucidate the changes to molecular pathways associated with 20E treatment, we carried out the RNA-seq assay. To do this, we treated H460 cells with 10 µM of 20E for 24 h. 

**Figure 4:**
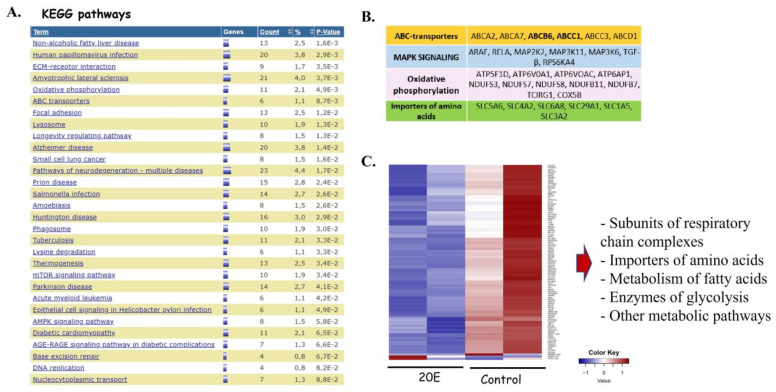
The analysis of genes which are down-regulated by the treatment of H460 cells with 10 µM of 20E for 24 h (RNA-seq data). (**A**) The DAVID gene clusterization predicted a number of molecular processes and diseases which may be affected by 20E. *p*-values indicate Fisher’s Exact *p*-values. (**B**) A table showing some 20E-suppressed genes. (**C**) Heatmap. Relative expression of 20E-suppressed genes involved in metabolic processes. Control—control samples; 20E—samples treated with 20E. Color Key indicates LogFold 2 values.

The analysis of differentially expressed genes showed that 20E may affect various processes in the cells (Figure 4A). All in all, we observed 619 down-regulated (Table S4) and 61 up-regulated (Table S5) genes.

Among the 20E-down-regulated genes in lung cancer cells, several of them are considered to be oncogenes, including Notch3 ([34]), HSF1 ([35]), mTOR ([36]), SOX12 ([37]), KLF16 (Kruppel-like factor 16, [38]), and others (Table S4). It is also important to note that 20E suppressed genes which code for ABC-transporters, including those conferring multidrug resistance ABCB6 and ABCC1 (MRP1), a cytokine TGF-β, MAPK signaling components, subunits of all respiratory chain complexes, and a number of amino acid importers (Figure 4A,B, Table S4). Additionally, several metabolic enzymes from various metabolic pathways may also be down-regulated according to the RNA-seq data (Figure 4B,C, Table S6). According to the data obtained, 20E may potentially affect glycolysis, oxidative phosphorylation, the metabolism of amino acids, and the biosynthesis of fatty acids and nucleotides.

Combining these data together with published reports from several groups, including ours, about 20E being able to modulate metabolism in muscle [1] and breast cancer cells [12], we decided to study its impact on the metabolic features of NSCLC cells.

### 3.4. 20E Down-Regulates Enzymes of Glycolysis and One-Carbon Metabolism

As metabolic rewiring is now recognized as one of the “hallmarks of cancer”, we decided to study how 20E may affect metabolic alterations in lung cancer cells. Enhanced glycolysis (“Warburg effect”) remains the main hub of cancer metabolism [39], whereas one-carbon (C1-) metabolism supplies one-carbon units critically important for nucleotide synthesis, methylation reactions, and for the generation of reducing cofactors, providing cancer cells with anabolic capacities [40].

H460, A549, and H1299 cell lines are frequently used as widely accepted cell models in the context of the metabolic rewiring of NSCLC [41,42,43]. To study the impact of 20E on the expression of genes involved in glycolysis and one-carbon metabolism, H460, A549, and H1299 NSCLC cells were treated with the extended concentration range of 20E (0.01–100 µM) for 48 h. In addition to genes coding for metabolic enzymes, we also analyzed the expression of two transcription factors which are deemed to be critical regulators of glycolysis and one-carbon metabolism: c-Myc [44,45,46] and ATF4, respectively [47,48,49]. We analyzed two key glycolytic genes encoding for hexokinase 2 (HK2) and lactate dehydrogenase A (LDHA). Regarding one-carbon metabolism, we assessed the expression of genes coding for all three steps of serine biosynthesis—PSAT1, PSPH, and PHGDH—as well as key mitochondrial enzymes of the folate cycle: SHMT2 and MTHFD2.

**Figure 5:**
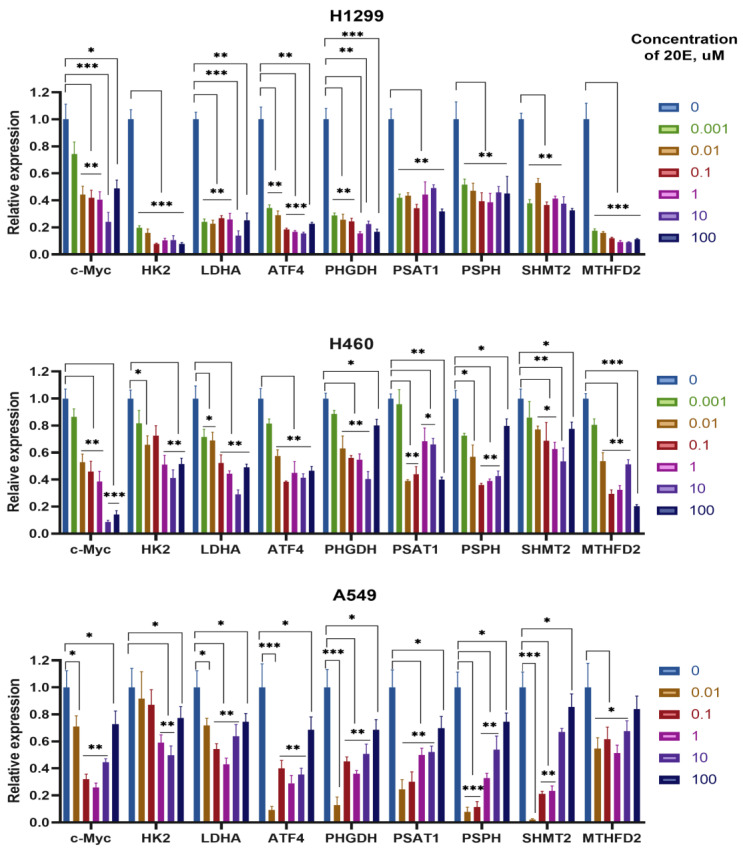
20E suppresses the expression of genes coding for enzymes of glycolysis, one-carbon metabolism, and their transcriptional regulators (Real-Time PCR). * *p* ≤ 0.05; ** *p* ≤ 0.01; *** *p* ≤ 0.001.

The Real-time PCR data have shown that even 0.01 µM of 20E may be sufficient to down-regulate the expression of all enzymes and their transcriptional regulators studied (Figure 5, Table S2). It should be noted that the degree of 20E-mediated suppression of gene expression depends on the cell line; however, in most cases 20E in concentrations of 0.01 or 0.1 µM was sufficient to down-regulate the expression.

**Figure 6:**
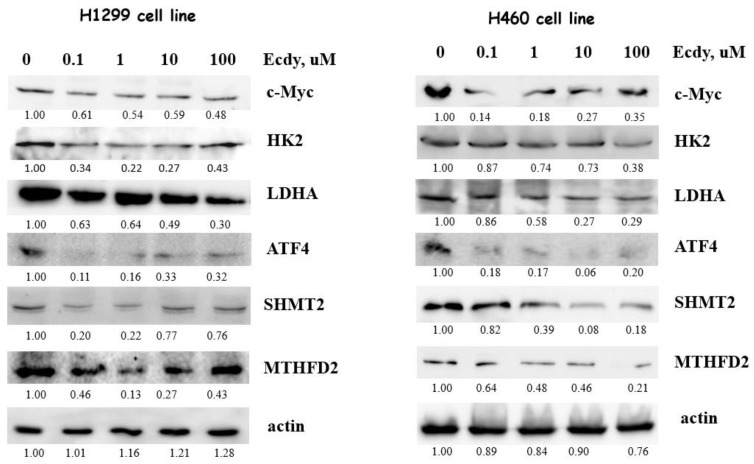
20E suppresses the expression of genes coding for enzymes of glycolysis, one-carbon metabolism, and their transcriptional regulators (Western-blot). The quantification was carried out using Image J software. For actin, the ratio of each sample/control sample is presented. For other proteins, the protein/actin ratio is shown.

Next, we used immunoblotting to assess the influence of 20E on the protein level of both the studied enzymes and their key regulators. For this purpose, we incubated H1299 and H460 cell lines with 0.1–100 µM of 20E for 3 days. Western-blot results confirmed the Real-time PCR data. As seen from Figure 6, 20E significantly suppressed HK2, LDHA, SHMT2, MTHFD2, c-Myc, and ATF4.

### 3.5. 20E Inhibits Glycolysis and Respiration in NSCLC Cell Lines

Having shown the suppression of key enzymes of glycolysis, as well as c-Myc and ATF4, we were then tasked to check the influence of 20E on glycolysis and respiration. Thus, we carried out energy profiling using the SeaHorse approach. NSCLC cell lines were treated with 0.1–100 µM of 20E for 48 h followed by the use of either SeaHorse MitoStress or Energy Profiling kits.

**Figure 7:**
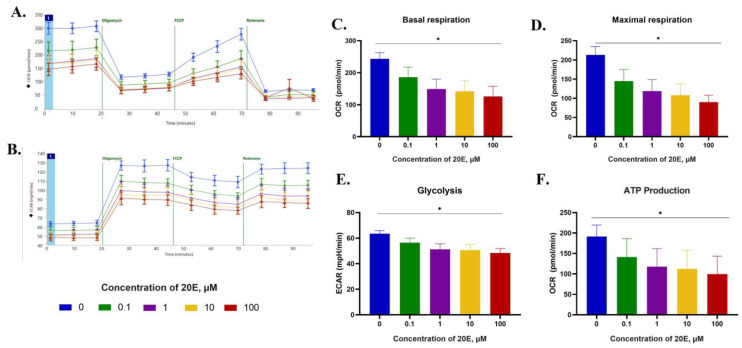
20E suppressed glycolysis and respiration in H460 cells. SeaHorse data. MitoStress test kit was used. (**A**,**B**)—OCR and ECAR plots. OCR—Oxygen Consumption Rate (shows respiration), ECAR—Extracellular Acidification Rate (shows glycolysis). (**C**) Basal respiration. (**D**) Maximal respiration. (**E**) Basal ECAR (glycolysis). (**F**) ATP production (in terms of respiration). * *p* ≤ 0.05.

We observed a significant 20E-mediated suppression of both glycolysis and respiration intensities in all cell lines (Figure 7, Figures S8 and S9, Table S2). In H460 cells, 20E inhibited respiration more pronouncedly (up to two times) than glycolysis (up to 30%) (Figure 7A–C,E). The maximal respiration capacity and ATP production rate calculated based on SeaHorse data were also both compromised (Figure 7D,F).

In H1299 cells, 20E in a concentration of 1 µM suppressed respiration and glycolysis by approximately 25% and 20%, respectively (Figure S8*,* Table S2). For A549 cells, the values were 30% and 25%, respectively (Figure S9*,* Table S2).

We also quantified the ATP production rate in H1299 and H460 cells treated with 0.1–100 µM of 20E for 3 days by using an ATP assay kit (Sigma). According to the results presented in Figure S10 and Table S2, 20E in all concentrations significantly reduced the level of ATP up to 2.5 times, which was consistent with SeaHorse, Real-time PCR, and Western-blot data on its impact on the glycolysis, respiration, and expression of glycolytic enzymes.

### 3.6. 20E Sensitizes Lung Cancer Cells to Metabolic Inhibitors

Both energy and one-carbon metabolism have been recognized as therapeutic targets in cancer therapy. As 20E has significantly suppressed both glycolysis and respiration, as well as the ATP content and expression of enzymes of one-carbon metabolism, we assessed whether 20E was able to modulate the susceptibility of cancer cells to the inhibitors of these respective metabolic processes.

To this end, we treated A549 and H460 cell lines with two inhibitors of glycolysis—2-deoxyglucose (2-DG, the inhibitor of HK2) and 3-brompyruvate (3-BP, the inhibitor of HK2, GAPDH, 3-PGK); one inhibitor of respiration (OxPhos)—metformin (MF, the inhibitor of the respiratory complex I); and two inhibitors of nucleotide and deoxyribonucleotide biosynthesis—5-fluouracil (5-FU, the inhibitor of thymidylate synthase-TYMS) and gemcitabine (Gemc, the inhibitor of deoxyribonucleotide reductase-dRNR). Notably, 2-DG, 3-BP, and MF have been used in multiple clinical trials, whereas 5-FU and gemcitabine are well known anticancer drugs.

We carried out the 72 h long treatment of cells with 20E or metabolic inhibitors individually or in several combinations. To study the mode of drug interaction, we used an online CompuSyn software (https://www.combosyn.com) which replicates the algorithms of Chou-Talalau [50]. 

**Figure 8:**
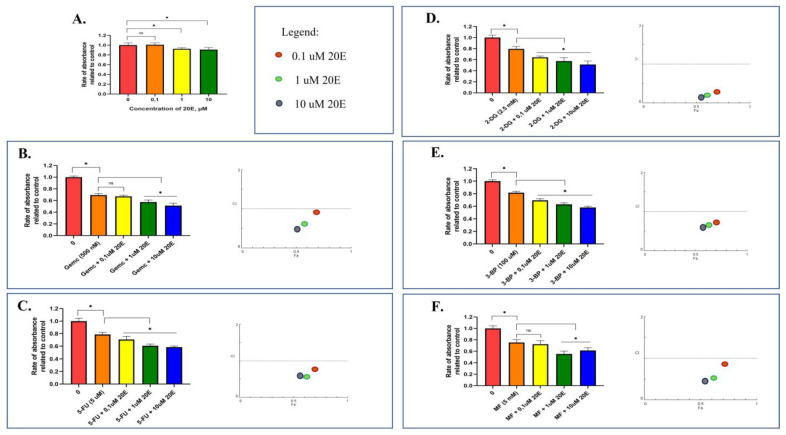
20E sensitizes A549 cells to inhibitors of glycolysis (2-DG—2-deoxyglucose, 3-BP—3-brompyruvate), oxidative phosphorylation (MF—metformin), and one-carbon metabolism (Gemc—gemcitabine, 5-FU—5-fluouracil). Cells were treated with 20E only (**A**), or in combination with (**B**) Gemc; (**C**) 5-FU; (**D**) 2-DG; (**E**) 3-BP; and (**F**) MF. MTT assay data are presented paired with Combination Index (CI) plots calculated using CompuSyn Software (https://www.combosyn.com). * *p* ≤ 0.05.

Figure 8 and Figure S11 and Table S2 show that 20E alone suppressed the proliferation of cancer cells to a maximum of 10% in both A549 and H460 cell lines. However, 20E in combination with 2-DG, MF, 3-BP, 5-FU, and Gemc significantly inhibited cancer cells relative to metabolic inhibitors alone. The quantitative analysis of drug interaction showed that 20E displayed synergy with all of the compounds tested. The Combinational Index (CI) values (Table 1) and CI plots in Figure 8 and Figure S11 reflect a significant synergistic interaction (CI < 1) of 20E with 2-DG, MF, 3-BP, 5-FU, and gemcitabine. 

These data mean that 20E can sensitize NSCLC cell lines to metabolic inhibitors.

### 3.7. The Increased Expression of 20E-Suppresed Metabolic Genes Is Associated with the Shortened Survival of Lung Cancer Patients

As stated earlier, increased glycolysis and one-carbon metabolism are known to be widely observed in various malignancies [20]. In this respect, we showed that 20E down-regulates the expression of genes coding for glycolytic enzymes HK2 and LDHA; enzymes of de novo serine biosynthesis PHGDH, PSAT1, and PSPH; key enzymes of mitochondrial folate cycle SHMT2 and MTHFD2; and their master-regulators c-Myc and ATF4 (CREB-2). To check whether the expression level of these genes was associated with prognosis in lung cancer patients, we used the online software KM Plotter (https://kmplot.com/analysis/, accessed on 15 January 2023) [27].

**Figure 9:**
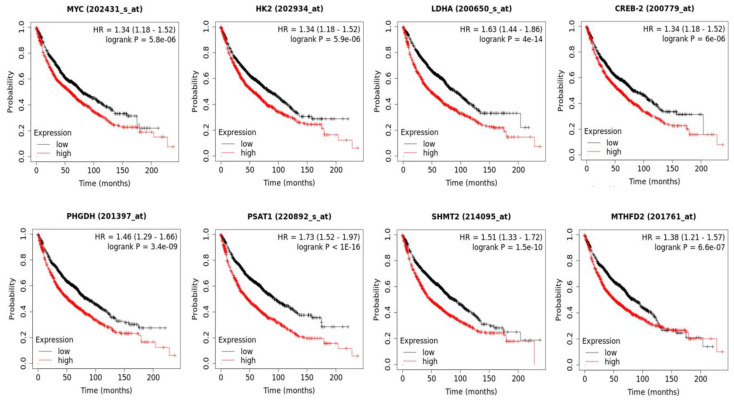
The high expression of 20E-suppressed genes coding for enzymes of glycolysis, one-carbon metabolism, and their transcriptional regulators c-Myc and ATF4 (CREB-2) are associated with the shortened survival of lung cancer patients. Kaplan–Meier plots were calculated using the online software KM Plotter (https://kmplot.com/analysis/, accessed on 15 January 2023).

The dataset consisted of 1925 lung cancer patients. The analysis has shown that with the exception of PSPH, the high expression of genes coding for all of these enzymes and their transcriptional regulators were strongly associated with a patient’s shortened survival (Figure 9). The corresponding medians of survival are demonstrated in Table S7.

### 3.8. 20E Suppressed the Expression of Genes Associated with Cancer Stem-like Cells

Cancer Stem Cells (CSCs) belong to a group of tumor cells with a multidirectional differentiation capacity, high self-renewal potential, and tumorigenicity [51]. Their presence is always associated with an increased incidence of metastasis, resistance to therapy, and tumor recurrence [52]. As the targeting of both glycolysis and respiration can be an effective strategy to eliminate CSCs, the 20E-induced down-regulation of these processes may theoretically suppress the CSCs’ population. Moreover, we observed the 20E-mediated suppression of colony formation in parallel with no obvious impact on cell cycle and apoptosis (Figure 3).

We carried out Real-Time PCR to determine whether 20E was able to affect the expression of CSCs-specific genes. For this purpose, we chose several common markers associated with lung cancer CSCs: aldehyde dehydrogenase (ALDH), CD44, Octamer-binding transcription factor 4 (Oct4), KIT proto-oncogene, receptor tyrosine kinase (c-Kit), and Nestin [51,52,53,54].

**Figure 10:**
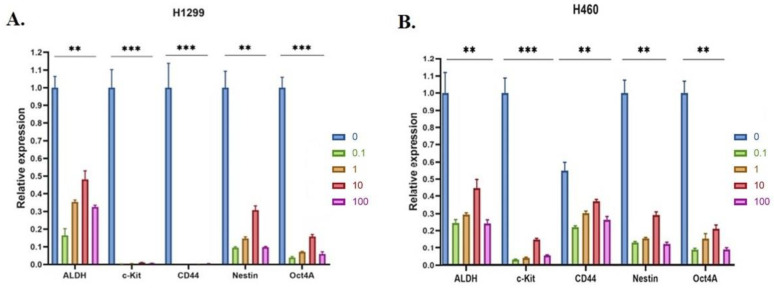
20E significantly suppresses the expression of markers of CSCs. Real-time PCR of (**A**) H1299 and (**B**) H460 cell lines, respectively. Results are shown as means ± SEM relative to control (DMSO-treated cells). One-way ANOVA with Dunnett’s test; *** *p* < 0.001, ** *p* < 0.01.

Figure 10 demonstrates that 20E treatment at doses of 0.1–100 µM significantly inhibited the expression of all of the CSC markers studied. The negative effect of 20E at doses of 0.1 and 1 µM was especially pronounced.

To verify these data, we established spheroids cultures of H1299 and H460 cell lines and treated them with the same doses of 20E. The results shown in Figure S12 and Table S2 proved these data. 

As CSCs represent a very important challenge for antineoplastic therapy, the 20E-mediated down-regulation of CSCs markers can be considered to be a very important anticancer feature.

## 4. Discussion

In the present research, we revealed the oncosuppressive role of 20E in NSCLC cell lines. As 20E has previously been shown to possess antioxidant capacities in both in vitro systems and non-cancer cells [30,31,32], firstly we checked whether this was the same for lung cancer cell models. Our results demonstrated that 20E displayed a strong antioxidant activity in concentrations of 0.1 µM in all three tumor cell lines tested, even after only 1.5 h of treatment. There have been several reports that 20E may suppress ROS in several in vitro-based systems.

However, it seems that the antioxidant properties of 20E are not limited by only the direct scavenger activity. Indeed, we showed that after 24 h of treatment, 20E increased the expression of genes coding for several key enzymes of antioxidant defense—Glutathione Peroxidases (GPX3, GPX4, GPX6), Glutathion Redustase (GSR), Glutathion Syntetase (GSS), Peroredoxines (PRDX1, PRDX5, PRDX6), Superoxiddismutases (SOD1, SOD2, SOD3), and others. Our data are consistent with the results of Gholipour et al. [32], who demonstrated a 20E-mediated increase in the activity of superoxide dismutase (SOD), catalase (CAT), Glutathione Peroxidase (GPx), and Glutathione Reductase (GRx) in neurons of an amyloid-beta-induced rat model of Alzheimer’s disease. It was also reported that 20E increased the amount of SOD in rat tongue after irradiation [33].

ROS are known to play a multifaced, opposing role in cancer, by favoring cancer onset at low doses on the one hand, and killing tumors at high doses on the other hand [29,55]. Usually, cancer cells have an increased level of intrinsic ROS [56]. The low and moderate increase in the levels of ROS may be a signal transducer for activate cell proliferation, migration, invasion, and angiogenesis, whereas a strong increase in ROS can induce the damage of proteins, nucleic acids, lipids, membranes, and organelles, which leads to cell death [57].

As ROS may play an opposite role in cancer, reducing or increasing intracellular ROS levels would be a potential strategy to prevent or treat cancer [29,58]. A number of natural compounds with strong antioxidant properties (quercetin [59], kaempferol [60], rutin [61], resveratrol [62]) have a well-established anticancer activity and are widely studied as potential antineoplastic therapeutics [63]. According to our results, 20E significantly suppressed the level of ROS in lung cancer cells, which may possibly decrease their oncogenic potential.

To further elucidate the possible mechanisms of 20E activity in lung cancer cells, we carried out an RNA-seq of 20E treated H460 cells. We observed the 20E-mediated down-regulation of about 50 genes considered to be oncogenes in NSCLC. Furthermore, a vast number of metabolic genes were also suppressed by 20E. Our further evaluation revealed that 20E inhibited the expression of key glycolytic genes HK2 and LDHA, as well as a number of genes coding for enzymes of serine biosynthesis (PSAT1, PHGDH, PSPH), folate cycle (SHMT2, MTHFD2), and their key transcriptional regulators—c-Myc and ATF4. In accordance with these data, 20E inhibited glycolysis, respiration, and ATP content. 

**Figure 11:**
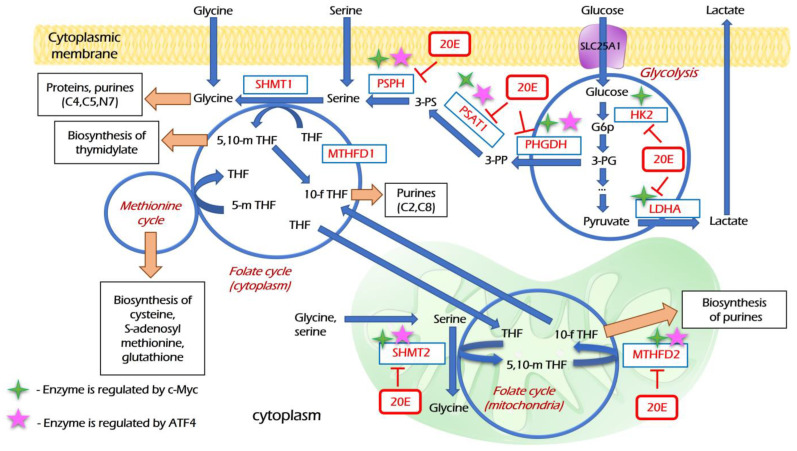
The hypothetical scheme suggesting how 20E can mediate the inhibition of glycolysis and one-carbon metabolism. 20E down-regulates glycolytic enzymes HK2 and LDHA. Serine and glycine are donors of C1-groups; they can be imported into the cell or synthesized *de novo* in three steps from 3-PG—the intermediate product of glycolysis by three enzymes, PHGDH, PSAT1, and PSPH. The expression of all of these enzymes is suppressed by 20E. Serine is converted to glycine by SHMT1 or SHMT2 in cytoplasmic and mitochondrial folate cycles, respectively, which results in the formation of 5,10-mTHF—the transmitter of C1-groups. MTHFD2 reduces m-THF to 10-fTHF as a part of folate cycle. SHMT2 and MTHFD2 are key enzymes of the mitochondrial folate cycle; they are down-regulated by 20E. 5,10-mTHF, 10-fTHF and glycine are donors of carbon groups for different biosynthetic processes—the biosynthesis of thymidylate, purine rings, S-adenosylmethionine, glutathione, etc.

Metabolic rewiring is considered one of the “hallmarks of cancer” [20]. Increased glycolysis and one-carbon metabolism are two principal metabolic alterations in neoplasia. Enhanced glycolysis implicates a myriad of molecular and functional processes to support cancer progression [64]. One-carbon metabolism supplies cancer cells with nucleotides, which limit cell divisions: SAM—the main donor of methyl groups; glutathione—an important factor of redox homeostasis; some amino acids; etc. [40,65]. Thus, it links glycolysis (the process of glucose assimilation) with different biosynthetic processes. The serine biosynthesis mediated by three enzymes—PSAT1, PHGDH, and PSAT1—“opens the gates” to one-carbon metabolism, whereas its central part—the folate cycle—mediates the re-distribution of one-carbon groups to acceptors [46] (Figure 11).

The up-regulation of glycolysis and one-carbon metabolism, and the increased expression of genes coding for corresponding enzymes, are usually observed in various neoplasms and are associated with metastasis, resistance to therapy, and poor prognosis. Our analysis of Kaplan–Meier plots derived from data on 1925 lung cancer patients clearly confirmed the strong oncogenic role of HK2, LDHA, PHGDH, PSAT1, SHMT2, MTHFD2, c-Myc, and ATF4 (CREB-2) in lung cancer. The high expression of genes coding for all of these enzymes and their transcriptional regulators is strongly associated with a shortened patient survival in different types of malignancies [66,67,68,69,70,71,72,73,74].

Different oncogenes and oncosupressors have a critical impact on these metabolic pathways [20,40,46,47,75,76]. Transcription factors c-Myc and ATF4 are two master-regulators of glycolysis, respiration, and one-carbon metabolism [44,45,46,49]. The inhibition of these cancer-associated metabolic pathways represents the attractive strategy of antineoplastic therapy. A number of glycolytic and OxPHOS inhibitors are currently being investigated in preclinical and clinical studies [20,77]. 2-DG, 3BP, and metformin sensitize cancer cells to different chemotherapeutic agents and irradiation [78,79]. In turn, one-carbon metabolism has been the target of anticancer therapy since the 1950s. Its inhibitors, methotrexate and its derivates 5-fluouracil and gemcitabine, are widely used in different chemotherapeutic schemes. The mitochondrial isoforms of folate cycle enzymes serine hydroxymethyl transferase and methylene-tetrahydrofolate reductase (SHMT2 and MTHFD2) are very important targets for drug development in cancer therapy [80].

We showed that 20E sensitizes NSCLC cell lines to some of these metabolic inhibitors, which may be therapeutically relevant (Figure 11). Reducing the dosage of metabolic inhibitors is very important since it helps to mitigate off-target effects. In turn, the inhibition of the energy and one-carbon metabolism in tumor cells usually sensitizes them to common therapeutics and radiotherapy [81]. Furthermore, it has been shown previously that 20E and its derivates modulate the resistance of cancer cells to genotoxic drugs and mitigate multidrug resistance [12,13,14,15,16,17,18,19].

CSCs are a subpopulation of tumor cells with the capacity for sustained self-renewal that, in turn, can not only drive tumor initiation but can also cause relapses, metastasis, and resistance to therapy [82,83]. Molecular markers of CSCs are not only used for diagnosis, but also have a therapeutic value because they are implicated in oncogenesis [84]. For lung cancer, ALDH, CD44, Oct4, c-kit, and Nestin were shown to be some of the most frequently observed CSCs markers which promote tumor development [53,54,85,86,87]. We showed that 20E significantly attenuated the level of their expression, which may be an important biomarker for the efficacy of antineoplastic therapy.

20E is a non-toxic substance for which pharmacokinetic studies have been carried out in both rodents and humans [8,9]. It has been evaluated in clinical trials to treat several disorders, including the severe consequences of COVID-19 (NCT04472728). The latter application of 20E in the treatment of post-COVID complications has reached stage III. According to observations of Dinan with colleagues [1], the maximal plasma concentration (C_max_) after the ingestion of 1400 mg of 20E was 710 ng/mL, which approximately corresponds to 1.5 µM. In our studies, 20E in a concentration of even 0.01 µM displayed a significant antioxidative effect, and therefore modulated the expression of metabolic genes. Importantly, when 20E was used at a concentration of 0.1 µM and higher, it had a clear impact on metabolism, sensitivity to metabolic inhibitors, and the expression of CSC markers.

It is interesting to note that we have frequently observed lesser effects for 100 uM of 20E as compared to the lower 20E concentrations in different experiments. The same phenomenon has been reported by other authors in different biological systems [88,89]. For instance, it was reported that low 20E concentration enhanced the growth of insect cells [88] and protein synthesis in mice myotubes [89], whereas a high concentration inhibited these processes. It seems that 20E possesses a bi-phasic (e.g., low-level stimulatory and high-level inhibitory) effect. We suggest that this may be associated with the nature of its interaction with specific receptors, because this type of kinetic is frequently observed for some hormones [90,91]. For example, it is already established that in muscle cells, 20E interacts with both MAS1 and estrogen receptor (ER), which both affect the 20E-modulated protein synthesis and myostatin expression [10]. We suggest that a pleiotropic activity of 20E in tissues of different origins cannot be determined by only one target. It seems that 20E may have several molecular targets, and the resulting effect is determined by their complex interaction.

According to the literature published to date, 20E possesses anti-oxidant, hepato- and cardioprotective, anti-inflammatory, hypolipidemic, hypoglycemic, and other pharmacological activities [1]. If our in vitro data on the anticancer effects of 20E can be translated into the clinic, this natural compound could potentially become a valuable adjuvant to decrease adverse effects of common anticancer therapies. Thus, further investigations regarding the antineoplastic properties of 20E are warranted. 

## Figures and Tables

**Table 1:** The quantitative analysis of drug interaction between 20E and metabolic inhibitors.

Dose 20E	Dose 2nd Compound	A549		H460	
		Effect	CI	Effect	CI
0.1 µM	2-DG (2.5 mM)	0.64	0.53	0.62	0.68
1 µM	2-DG (2.5 mM)	0.57	0.42	0.56	0.59
10 µM	2-DG (2.5 mM)	0.51	0.37	0.49	0.51
0.1 µM	MF (5 mM)	0.72	0.85	0.82	0.59
1 µM	MF (5 mM)	0.61	0.52	0.81	0.57
10 µM	MF (5 mM)	0.55	0.44	0.79	0.52
0.1 µM	3-BP (100 µM)	0.69	0.71	0.71	0.69
1 µM	3-BP (100 µM)	0.63	0.63	0.65	0.59
10 µM	3-BP (100 µM)	0.58	0.61	0.56	0.48
0.1 µM	Gemc (500 nM)	0.67	0.91	0.75	0.6
1 µM	Gemc (500 nM)	0.57	0.59	0.75	0.6
10 µM	Gemc (500 nM)	0.51	0.48	0.64	0.37
0.1 µM	5-FU (5 µM)	0.7	0.75	0.69	0.76
1 µM	5-FU (5 µM)	0.6	0.56	0.64	0.68
10 µM	5-FU (5 µM)	0.58	0.57	0.64	0.68

CI—Combinational Index.

## Data Availability

The RNA-seq raw data are deposited in the SRA database https://www.ncbi.nlm.nih.gov/sra/PRJNA947934 (BioProject ID: PRJNA947934).

## Supplementary Materials

The following supporting information can be downloaded at: https://www.mdpi.com/article/10.3390/metabo13050656/s1, Figure S1. The 24 h treatment with 20E suppresses ROS in NSCLC cell lines. A–C—diagrams of relative DCDFA fluorescence for H1299, H460, and A549 cells treated with 0.1–100 µM of 20E. Y-axis shows the degree of fluorescence intensity of 20E treated cells relative to fluorescence of control cells. D–F—flow cytometry plots for DCDFA fluorescence; ‘median’ of the peak for the control sample is showed by vertical bar. *** *p* ≤ 0.001; **** *p* ≤ 0.0001. Figure S2. Cell cycle assay of H460 cells treated with 20E for 48 h. A. The diagram shows percentage of cells in G0/G1, S, and G2/M phases. B. Quantitative data. C. Flow cytometry plots. Figure S3. Cell cycle assay of A549 cells treated with 20E for 48 h. A. The diagram shows percentage of cells in G0/G1, S, and G2/M phases. B. Quantitative data. C. Flow cytometry plots. Figure S4. Cell cycle assay of H1299 cells treated with 20E for 48 h. A. The diagram shows percentage of cells in G0/G1, S, and G2/M phases. B. Quantitative data. C. Flow cytometry plots. Figure S5. Results of Annexin V test of H460 cells treated with 20E for 48 h. A. Flow cytometry plots. B. Table showing the percentage of dead cells. Figure S6. Results of Annexin V test of A549 cells treated with 20E for 48 h. A. Flow cytometry plots. B. Table showing the percentage of dead cells. Figure S7. Results of Annexin V test of H1299 cells treated with 20E for 48 h. A. Flow cytometry plots. B. Table showing the percentage of dead cells. Figure S8. 20E suppressed glycolysis and respiration in H1299 cells. SeaHorse data. Energy Phenotype test kit was used. A–D—OCR and ECAR plots. OCR—Oxygen Consumption Rate (shows respiration), ECAR—Extracellular Acidification Rate (shows glycolysis). E. Energetic map. * *p* ≤ 0.05. Figure S9. 20E suppressed glycolysis and respiration in A549 cells. SeaHorse data. Energy Phenotype test kit was used. A–D—OCR and ECAR plots. OCR—Oxygen Consumption Rate (shows respiration), ECAR—Extracellular Acidification Rate (shows glycolysis). E. Energetic map. * *p* ≤ 0.05. Figure S10. 20E reduced ATP content in A. H1299 and B. H460 cells. ATP assay kit (Sigma) was used. * *p* ≤ 0.05; ** *p* ≤ 0.01; *** *p* ≤ 0.001; **** *p* ≤ 0.0001. Figure S11. 20E sensitizes H460 cells to inhibitors of glycolysis (2-DG—2-deoxyglucose, 3-BP—3-brompyruvate), oxidative phosphorylation (MF—metformin), and one-carbon metabolism (Gemc—gemcitabine, 5-FU—5-fluouracil). Cells were treated with 20E only (A.) or in combination with B. Gemc; C. 5-FU; D. 2-DG; E. 3-BP; F. MF. MTT assay data are presented in pair with Combination Index (CI) plots calculated using CompuSyn Software (https://www.combosyn.com). * *p* ≤ 0.05. Figure S12. 48 h treatment with 20E significantly suppressed expression of markers of CSCs in spheroid culture. A. H460 cell line. B. H1299 cell line. Real-time PCR. Results are shown as means ± SEM relative to control (DMSO-treated cells). One-way ANOVA with Dunnett’s test; *** *p* < 0.001. Table S1. Sequences of primers used for Real-Time PCR. Table S2. Additional quantitative data for experiments. Table S3. The change in gene expressions associated with antioxidant response after the treatment of H1299 and H460 cells with 20E for 24 h (Qiagen RT² Profiler™ PCR Array Human Oxidative Stress kit). The Fold Regulations display a value of the normalized gene expression in 20E-treated cells which divided the normalized gene expression in the control (DMSO-treated) cells. Table S4. The list of down-regulated genes upon 24 h treatment of H460 cells with 20E (*p* ≤ 0.05). Table S5. The list of up-regulated genes upon 24 h treatment of H460 cells with 20E (*p* ≤ 0.05). Table S6. The list of down-regulated genes associated with metabolic pathways upon 24 h treatment of H460 cells with 20E (*p* ≤ 0.05). Table S7. Median values (gathered over a month) of lung cancer patients’ survival with low and high expression of studied genes. Values were calculated using the online software KM Plotter (https://kmplot.com/analysis/).
