# Supplementary material for: 20-Hydroxyecdysone Confers Antioxidant and Antineoplastic Properties in Human Non-Small Cell Lung Cancer Cells
Source: Metabolites. 2023 May 15;13(5):656. doi: 10.3390/metabo13050656 (PMC10223942; doi:10.3390/metabo13050656)
Supplement: Supplementary file 1 [file metabolites-13-00656-s001.zip › Supplementary Tables.zip!/Table S1.pdf]

Primers used for Real-Time PCR:

ATF4_F: GGAGATAGGAAGCCAGACTACA;
ATF4_R: GGCTCATA-CAGATGCCACTATC;
PSAT1__F: CAGGAAGGTGTGCTGACTATG;
PSAT1_R: CCCAAGTTTAGGGTGAACGATA;
PSPH_F: AGCTGTTCCAGACAACTATAAGG;
PSPH_HS_R: CTTCCTGCTTTCAAGCCAATG;
PHGDH_F: GATCTGGCCTCTCTGTGATTT;
PHGDH_R: GGGCAAAGGTGTTGTCATTC;
SHMT2_F: AGGTCAGGGCTCATCTTCTA;
SHMT2_R: ATGGGAACACGGCAAAGT;
MTHFD2_F: AGCTTTACCTCTTGGCAGTATC;
MTHFD2_R: CTG-TAATTCCCAGCGTCTAGTG;
HK2_F: AAGGCTTCAAGGCATCTG;
HK2_R: GCCAGGTCCTTCACTGTCTC;
LDHA_F: AGATTCCAGTGTGCCTGTATG;
LDHA_R: ACCTCTTTCCACTGTTCCTTATC;
Myc_F: CTCCTCCTCGTCGCAGTAGA;
Myc_R: GCTGCTTAGACGCTGGATTT;
β-Actin_F: GGATCAGCAAGCAGGAGTATG;
β-Actin_R: AGAAAGGGTGTAACGCAACTAA;
Oct4_F: TCGCAAGCCCTCATTTCACC;
Oct4_R: GCCAGGTCCGAGGATCAAC;
Nestin_F: GTAGCTCCCAGAGAGGGGAA;
Nestin_R: CTCTAGAGGGCCAGGGACTT;
c-Kit_F: GATGGCACCTGAAAGCATTT;
c-Kit_R: TCCGGAAGCCTTCCTTGATC;
CD44_F: AAGGTGGAGCAAACACAACC;
CD44_R: ACTGCAATGCAAACTGCAAG;
ALDH_F: GTTGTCAAACCAGCAGACCA;
ALDH_R: CTGTAGGCCCATAACCAGGA.
