# Supplementary material for: 20-Hydroxyecdysone Confers Antioxidant and Antineoplastic Properties in Human Non-Small Cell Lung Cancer Cells
Source: Metabolites. 2023 May 15;13(5):656. doi: 10.3390/metabo13050656 (PMC10223942; doi:10.3390/metabo13050656)
Supplement: Supplementary file 1 [file metabolites-13-00656-s001.zip › Supplementary Tables.zip!/Table S2.pdf]

Figure 1 Supplementary Data. The 1.5 hours of treatment with 20E suppresses ROS in NSCLC cell lines. N=4

A549

0		0,1		1		10		100	
Mean	SEM	Mean	SEM	Mean	SEM	Mean	SEM	Mean	SEM
1	2e-002	0.748036	0.029524	0.702656	0.036697	0.695774	0.025364	0.829809	0.017109

H1299

0		0,1		1		10		100	
Mean	SEM	Mean	SEM	Mean	SEM	Mean	SEM	Mean	SEM
1	2e-002	0.77238	0.01119	0.713652	0.021327	0.573844	0.023590	0.638733	0.031027

H460

0		0,1		1		10		100	
Mean	SEM	Mean	SEM	Mean	SEM	Mean	SEM	Mean	SEM
1	1e-002	0.790966	0.036352	0.655795	0.027595	0.647772	0.025799	0.708875	0.020095

Figure 3 Supplementary Data. 20E inhibits the growth of NSCLC cell lines. A-C. MTT data for H1299, A549 and H460 cell lines. N=8. D. Colony formation assay. N=3.

3A.

0		0.1		1		10		100	
Mean	SEM	Mean	SEM	Mean	SEM	Mean	SEM	Mean	SEM
1.0000	0.0250	0.912000	0.034000	0.894000	0.033000	0.875000	0.032000	0.912000	0.025000

3B.

0		0.1		1		10		100	
Mean	SEM	Mean	SEM	Mean	SEM	Mean	SEM	Mean	SEM
1	9e-003	0.821244	0.013591	0.905391	0.012946	0.853546	0.021369	0.835613	0.018053

3C.

0		0,1		1		10		100	
Mean	SEM	Mean	SEM	Mean	SEM	Mean	SEM	Mean	SEM
1	2e-002	0.97	0.02	0.887	0.041	0.917	0.014	0.900	0.031

3D.

H1299

0		0.1		1		10		100	
Mean	SEM	Mean	SEM	Mean	SEM	Mean	SEM	Mean	SEM
153.5	1.1	134.6667	3.3599	135.3333	5.2747	138.6667	1.5776	145	2

A549

0		0.1		1		10		100	
Mean	SEM	Mean	SEM	Mean	SEM	Mean	SEM	Mean	SEM
153.5	1.1	134.6667	3.3599	133.6667	3.8064	138.6667	1.5776	145	2

H460

0		0.1		1		10		100	
Mean	SEM	Mean	SEM	Mean	SEM	Mean	SEM	Mean	SEM
198.6667	4.6571	188.6667	7.2541	160.6667	4.7981	171	2	158.3333	3.9441

Figure 5 Supplementary Data. 20E suppresses the expression of genes coding for enzymes of glycolysis, one-carbon metabolism, and their transcriptional regulators (Real-Time PCR). N=3.

H1299

	0		0.001		0.01		0.1		1		10		100	
	Mean	SEM	Mean	SEM	Mean	SEM	Mean	SEM	Mean	SEM	Mean	SEM	Mean	SEM
c-Myc	1	1e-001	0.74243	0.08971	0.44322	0.06094	0.41888	0.05666	0.40636	0.05850	0.24159	0.06978	0.48892	0.06164
HK2	1	7e-002	0.19658	0.01609	0.15986	0.02830	0.07775	0.00580	0.10486	0.01435	0.10671	0.03221	0.07876	0.00905
LDHA	1	5e-002	0.24007	0.02232	0.22637	0.02750	0.26896	0.01892	0.25854	0.04609	0.13830	0.03595	0.25385	0.05281
ATF4	1	9e-002	0.34440	0.02222	0.29077	0.03077	0.18586	0.00890	0.16701	0.00994	0.15472	0.01011	0.22742	0.01029
PHGDH	1	8e-002	0.28797	0.01844	0.25769	0.03976	0.24502	0.02222	0.15598	0.01214	0.22462	0.02177	0.16662	0.02132
PSAT1	1	8e-002	0.41818	0.02833	0.43414	0.02238	0.34129	0.02899	0.44394	0.09353	0.49063	0.02492	0.31713	0.01973
PSPH	1	1e-001	0.51772	0.03986	0.47051	0.05683	0.39488	0.06163	0.38548	0.06670	0.45815	0.04434	0.45182	0.12563
SHMT2	1	5e-002	0.37845	0.02805	0.52956	0.03217	0.36623	0.02222	0.41424	0.01749	0.37586	0.05047	0.32524	0.01571
MTHFD2	1	1e-001	0.17582	0.01343	0.15934	0.01038	0.12027	0.00699	0.09296	0.00998	0.08960	0.00423	0.11270	0.00472

H460

	0		0.001		0.01		0.1		1		10		100	
	Mean	SEM	Mean	SEM	Mean	SEM	Mean	SEM	Mean	SEM	Mean	SEM	Mean	SEM
c-Myc	1	7e-002	0.864870	0.058400	0.52847	0.06130	0.45967	0.07625	0.386270	0.074610	0.08615	0.01326	0.141340	0.028740
HK2	1	6e-002	0.816360	0.095780	0.65738	0.06734	0.72645	0.07296	0.511899	0.067170	0.41246	0.05973	0.515169	0.043560
LDHA	1	9e-002	0.716450	0.056470	0.68957	0.06132	0.52211	0.06144	0.444090	0.020600	0.29159	0.03165	0.491990	0.022150
ATF4	1	7e-002	0.815080	0.033360	0.57552	0.04516	0.38479	0.00469	0.449630	0.084020	0.41343	0.02998	0.464640	0.032670
PHGDH	1	4e-002	0.885970	0.026640	0.63031	0.09329	0.56168	0.01712	0.547050	0.043460	0.40359	0.05575	0.800960	0.045960
PSAT1	1	3e-002	0.957390	0.109280	0.39074	0.00926	0.44049	0.05624	0.684420	0.097930	0.66089	0.04480	0.399070	0.019670
PSPH	1	6e-002	0.726700	0.015950	0.56890	0.08712	0.35977	0.01252	0.390300	0.014180	0.42589	0.03722	0.797030	0.052140
SHMT2	1	7e-002	0.859469	0.118990	0.77096	0.02568	0.68762	0.13549	0.626780	0.049400	0.53448	0.10041	0.774410	0.051510
MTHFD2	1	4e-002	0.805390	0.044160	0.53736	0.06180	0.29590	0.02729	0.324500	0.031120	0.51245	0.03541	0.203000	0.012980

A549

	0		0.01		0.1		1		10		100	
	Mean	SEM	Mean	SEM	Mean	SEM	Mean	SEM	Mean	SEM	Mean	SEM
c-Myc	1	1e-001	0.71090	0.07900	0.32060	0.03670	0.25980	0.03134	0.44560	0.02560	0.72850	0.09674
HK2	1	1e-001	0.91788	0.19880	0.87094	0.11290	0.59174	0.05670	0.49760	0.06924	0.77430	0.08304
LDHA	1	1e-001	0.71720	0.05449	0.54321	0.04000	0.42960	0.04660	0.63950	0.08530	0.74504	0.06110
ATF4	1	2e-001	0.09244	0.02559	0.39905	0.06020	0.28925	0.05954	0.35443	0.04697	0.68524	0.09585
PHGDH	1	1e-001	0.12820	0.06053	0.45210	0.03302	0.35975	0.02490	0.50664	0.07277	0.68626	0.07527
PSAT1	1	1e-001	0.24543	0.07171	0.30035	0.07394	0.49962	0.04940	0.52062	0.04533	0.69842	0.08587
PSPH	1	1e-001	0.07872	0.03449	0.11321	0.04099	0.32960	0.03516	0.53950	0.09953	0.74504	0.06533
SHMT2	1	1e-001	0.02090	0.00681	0.21106	0.01969	0.23298	0.03716	0.66986	0.02628	0.85469	0.09674
MTHFD2	1	2e-001	0.54667	0.08123	0.61766	0.08847	0.51395	0.05834	0.67692	0.07547	0.83999	0.09578

Figure 7 Supplementary Data. 20E suppressed glycolysis and respiration in H460 cells. SeaHorse data. MitoStress test kit was used. A. and B. – OCR and ECAR plots. OCR – Oxygen ConsμMption Rate (shows respiration), ECAR – Extracellular Acidification Rate (shows glycolysis). C. Basal respiration. D. Maximal respiration. E. Basal ECAR (glycolysis). F. ATP production (in terms of respiration).

Glycolysis (ECAR); Basal respiration (OCR). N=5.

	**ECAR (mpH/min)**		**OCR (pmol/min)**	
**Group**	**Value**	**StandardMean**	**Value**	**StandardMean**
0	63,44030	2,42249	299,03845	21,41248
0.1	56,44988	3,57530	214,79247	32,17472
1	51,27338	4,37246	166,21213	29,64260
10	50,60178	4,53704	165,71639	34,75343
100	48,47167	3,36740	145,26754	22,87614

Figure 8 Supplementary Data. 20E sensitizes A549 cells to inhibitors of glycolysis (2-DG – 2-deoxyglucose, 3-BP – 3-brompyruvate), oxidative phosphorylation (MF - metformin), and one-carbon me-tabolism (Gemc – gemcitabine, 5-FU – 5-fluouracil). Cells were treated with 20E only (A.) or in combination with: B. Gemc; C. 5-FU; D. 2-DG; E. 3-BP; F. MF. MTT assay data are presented paired with Combination Index (CI) plots calculated using CompuSyn Software (https://www.combosyn.com). N=8.

	Result	SEM
0	1	0,026512
0,1uM 20E	0,954598	0,026496
1uM 20E	0,894253	0,003073
10uM 20E	0,939655	0,033152
0	1	0,026512
5-FU (5 uM)	0,793678	0,042616
5-FU + 0,1uM 20E	0,641379	0,009088
5-FU + 1uM 20E	0,691379	0,054206
5-FU + 10uM 20E	0,648851	0,014874
0	1	0,026512
MF (5 mM)	0,941954	0,019203
MF + 0,1uM 20E	0,783333	0,020822
MF + 1uM 20E	0,816092	0,043691
MF + 10uM 20E	0,82069	0,017235
0	1	0,026512
Gemc (500 nM)	0,844253	0,042188
Gemc + 0,1uM 20E	0,745977	0,013904
Gemc + 1uM 20E	0,754598	0,031055
Gemc + 10uM 20E	0,641379	0,009088
0	1	0,026512
2-DG (2.5 mM)	0,768391	0,040193
2-DG + 0,1 uM 20E	0,624138	0,061293
2-DG + 1uM 20E	0,565517	0,064859

2-DG + 10uM 20E	0,493678	0,048051
0	1	0,026512
3-BP (100 uM)	0,828161	0,039037
3-BP + 0,1uM 20E	0,714368	0,031633
3-BP + 1uM 20E	0,657471	0,044874
3-BP + 10uM 20E	0,564943	0,050763

Figure 10 Supplementary Data. 20E significantly suppresses the expression of markers of CSCs. Real-time PCR of A. H1299, B. H460 cell lines, respectively. Results are shown as means ± SEM relative to control (DMSO-treated cells). N=3.

10A.

	0		0,1		1		10		100	
	Mean	SEM	Mean	SEM	Mean	SEM	Mean	SEM	Mean	SEM
ALDH	1	6e-002	0.16470	0.03773	0.35385	0.01069	0.48179	0.04771	0.32492	0.01009
c-Kit	1	1e-001	0.00247	0.00092	0.00484	0.00060	0.01072	0.00117	0.00649	0.00134
CD44	1	1e-001	0.00022	0.00002	0.00026	0.00005	0.00065	0.00015	0.00331	0.00241
Nestin	1	9e-002	0.09502	0.00527	0.14714	0.00923	0.30791	0.02344	0.09840	0.00341
Oct4A	1	6e-002	0.03942	0.00566	0.07144	0.00276	0.15825	0.01198	0.05965	0.01195

10B.

	0		0,1		1		10		100	
	Mean	SEM	Mean	SEM	Mean	SEM	Mean	SEM	Mean	SEM
ALDH	3.80673	0.45032	0.92943	0.07223	1.11585	0.03767	1.69825	0.19273	0.91756	0.08027
c-Kit	3.80673	0.32967	0.11692	0.01257	0.15726	0.01423	0.56354	0.02554	0.20842	0.01497
CD44	2.08399	0.18611	0.83526	0.03026	1.14357	0.04690	1.40917	0.03983	1.00000	0.07139
Nestin	3.80673	0.28313	0.49553	0.02185	0.58697	0.02204	1.10682	0.06600	0.46550	0.03583
Oct4A	3.80673	0.25962	0.33549	0.03190	0.58165	0.11141	0.80000	0.08423	0.34425	0.03512

Figure S1 Data. The 24 hours of treatment with 20E suppresses ROS in NSCLC cell lines. A-C – diagrams of rela-tive DCDFA fluorescence for H1299, H460 and A549 cells treated with 0.1-100 µM of 20E. Y-axis shows the degree of fluorescence intensity of 20E treated cells relative to fluorescence of control cells. D-F – flow cytometry plots for DCDFA fluorescence, 'median' of the peak for the control sample is showed by vertical bar. N=8.

H1299

0		0.1		1		10		100	
Mean	SEM	Mean	SEM	Mean	SEM	Mean	SEM	Mean	SEM
1	3e-002	0.856887	0.019883	0.796743	0.016335	0.754724	0.033375	0.831065	0.039934

H460

0		0.1		1		10		100	
Mean	SEM	Mean	SEM	Mean	SEM	Mean	SEM	Mean	SEM
1	2e-002	0.801229	0.019601	0.7941	0.0198	0.746198	0.004798	0.717739	0.008864

A549

0		0.1		1		10		100	
Mean	SEM	Mean	SEM	Mean	SEM	Mean	SEM	Mean	SEM
1	3e-002	0.858987	0.035520	0.828355	0.022932	0.701004	0.021312	0.765042	0.014818

Figure S8 Data. 20E suppressed glycolysis and respiration in H1299 cells. SeaHorse data. Energy Phenotype test kit was used. A-D – OCR and ECAR plots. OCR – Oxygen Consumption Rate (shows respiration), ECAR – Extracellular Acidification Rate (shows glycolysis). E. Energetic map.

	**ECAR (mpH/min)**		**OCR (pmol/min)**	
**Group**	**Value**	**StandardMean**	**Value**	**StandardMean**
0	41,08417	2,93233	90,56009	8,23473
1	32,28381	6,46456	65,60394	13,87788
10	29,58232	3,17483	53,84928	8,37005
100	28,12036	4,00277	56,88195	7,20560

Figure S9 Data. 20E suppressed glycolysis and respiration in A549 cells. SeaHorse data. Energy Phenotype test kit was used. A-D – OCR and ECAR plots. OCR – Oxygen Consumption Rate (shows respiration), ECAR – Extracellular Acidification Rate (shows glycolysis). E. Energetic map.

	**ECAR (mpH/min)**		**OCR (pmol/min)**		
**Group**	**Value**	**StandardMean**	**Value**	**StandardMean**	
0	52,03607	1,58128	196,89375	12,75162	
1	39,98037	4,26154	157,29196	24,96845	
10	38,49924	5,97182	136,17414	31,32935	
100	18,44500	3,30770	60,40648	14,75454	

Figure S10 Data. 20E reduced ATP content in A. H1299 and B. H460 cells. ATP assay kit (Sigma) was used. N=3.

H460

0		0.1		1		10		100	
Mean	SD	Mean	SD	Mean	SD	Mean	SD	Mean	SD
1.000	0.006	0.464912	0.003899	0.830409	0.008066	0.608187	0.009893	0.918129	0.003386

H1299

0		0.1		1		10		100	
Mean	SD	Mean	SD	Mean	SD	Mean	SD	Mean	SD
1.000000	0.060016	0.584778	0.019480	0.597589	0.024809	0.502638	0.009136	0.676714	0.008959

Figure S11 Data. 20E sensitizes A549 cells to inhibitors of glycolysis (2-DG – 2-deoxyglucose, 3-BP – 3-brompyruvate), oxidative phosphorylation (MF - metformin), and one-carbon metabolism (Gemc – gemcitabine, 5-FU – 5-fluouracil). Cells were treated with 20E only (A.) or in combination with: B. Gemc; C. 5-FU; D. 2-DG; E. 3-BP; F. MF. MTT assay data are presented in pair with Combination Index (CI) plots calculated using CompuSyn Software (https://www.combosyn.com). MTT assay, A549 cells. N=8.

		Results	SEM
0		1	0,045234
0,1uM 20E		1,00869	0,0368
1uM 20E		0,924131	0,025846
10uM 20E		0,909425	0,042957
0		1	0,045234
2-DG (2.5 mM)		0,794118	0,046815
2-DG + 0,1 uM 20E		0,642714	0,024135
2-DG + 1uM 20E		0,575869	0,058602
2-DG + 10uM 20E		0,513369	0,061963
0		1	0,045234
MF (5 mM)		0,753008	0,049504
MF + 0,1uM 20E		0,722594	0,061876
MF + 1uM 20E		0,555481	0,048898
MF + 10uM 20E		0,613971	0,048427
0		1	0,045234
5-FU (5 uM)		0,78643	0,034502
5-FU + 0,1uM 20E		0,707553	0,049998
5-FU + 1uM 20E		0,606283	0,028465
5-FU + 10uM 20E		0,584559	0,018668
0		1	0,019779
Gemc (500 nM)		0,693387	0,028011
Gemc + 0,1uM 20E		0,671935	0,020055
Gemc + 1uM 20E		0,573419	0,036358

Gemc + 10uM 20E		0,513419	0,04201
0		1	0,022783
3-BP (100 uM)		0,817698	0,02409
3-BP + 0,1uM 20E		0,694127	0,026296
3-BP + 1uM 20E		0,631497	0,021802
3-BP + 10uM 20E		0,580301	0,02051

Figure S12 Data. 48 h treatment with 20E significantly suppressed expression of markers of CSCs in spheroid culture. A. H460 cell line. B. H1299 cell line. Real-time PCR. Results are shown as means ± SEM relative to control (DMSO-treated cells). N=3.

12A. H460

	0		1	
	Mean	SEM	Mean	SEM
CD44	1	3e-002	0.18039	0.01207
c-Kit	1	1e-001	0.03869	0.00203
ALDH1	1	3e-002	0.16189	0.00466
Nestin	1	7e-002	0.07154	0.03267
Oct4	1	2e-001	0.09077	0.00735

12B. H1299

	0		1	
	Mean	SEM	Mean	SEM
c-Kit	1	1e-001	0.15730	0.09460
ALDH1	1	1e-001	0.13835	0.01891
Nestin	1	2e-001	0.01021	0.00161
Oct4	1	1e-001	0.09979	0.00013
