# Supplementary material for: 20-Hydroxyecdysone Confers Antioxidant and Antineoplastic Properties in Human Non-Small Cell Lung Cancer Cells
Source: Metabolites. 2023 May 15;13(5):656. doi: 10.3390/metabo13050656 (PMC10223942; doi:10.3390/metabo13050656)
Supplement: Supplementary file 1 [file metabolites-13-00656-s001.zip › Supplementary Tables.zip!/Table S7.pdf]

Table S6 Data. Median values (gathered over a month) of lung cancer patient's survival with low and high expression of studied genes. Values were calculated using the online software KM Plotter (https://kmplot.com/analysis/).

	**Low expression cohort (months)**	**High expression cohort (months)**	**p-value**
**c-Myc**	79.5	59	5.8e-6
**Hk2**	85	56.5	5.9e-6
**LDHA**	90	47.77	4.0e-14
**ATF4**	81.1	57	6.0e-6
**PHGDH**	85	52	3.4e-9
**PSAT1**	93	46.7	<1e-16
**SHMT2**	89	48	1.5e-10
**MTHFD2**	85	52	6.6e-7
